# Association between ventilatory efficiency, oxygen uptake, and Glittre-ADL test results in patients with chronic heart failure: a preliminary study

**DOI:** 10.1186/s13104-021-05479-x

**Published:** 2021-02-16

**Authors:** Hebert Olímpio Júnior, Agnaldo José Lopes, Fernando Silva Guimarães, Sergio Luiz Soares Marcos da Cunha Chermont, Sara Lúcia Silveira de Menezes

**Affiliations:** 1 Medical Sciences Post-Graduation Programme, School of Medical Sciences, State University of Rio de Janeiro (UERJ), Av. Prof. Manoel de Abreu, 444, 2º andar, Vila Isabel, Rio de Janeiro, 20550-170 Brazil; 2 Rehabilitation Sciences Post-Graduation Programme, Augusto Motta University Center (UNISUAM), Rua Dona Isabel, 94, Rio de Janeiro, Bonsucesso 21032-060 Brazil; 3 Department of Physiotherapy, Federal University of Rio de Janeiro (UFRJ), Rua Prof. Rodolpho Paulo Rocco, Cidade Universitária, Rio de Janeiro, 25521941-590 Brazil; 4 Post-Graduate Programme in Cardiovascular Sciences, Federal Fluminense University (UFF), Rua Marquês Do Paraná, 303, 4º andar, Niterói, 24033-900 Brazil

**Keywords:** Chronic heart failure, Functional capacity, Glittre-ADL test

## Abstract

**Objective:**

The Glittre-ADL test (GA-T) is a functional capacity test that stands out for encompassing multiple tasks similar to activities of daily living. As ventilatory efficiency is one of the variables valued in the prognosis of chronic heart failure (CHF), this study aimed to evaluate associations between functional capacity and ventilatory variables in patients with CHF during the GA-T.

**Results:**

Eight patients with CHF and New York Heart Association (NYHA) functional classification II–III underwent the GA-T coupled with metabolic gas analysis to obtain data by means of telemetry. The median total GA-T time was 00:04:39 (00:03:29–00:05:53). Borg dyspnoea scale scores before and after the GA-T were 2 (0–9) and 3 (1–10), respectively (*P* = 0.011). The relationship between the regression slope relating minute ventilation to carbon dioxide output (VE/VCO_2_ slope) was correlated with the total GA-T time (r_s_ = 0.714, *P* = 0.047) and Borg dyspnoea score (r_s_ = 0.761, *P* = 0.028). The other ventilatory variables showed no significant correlations. Our results suggest that the total GA-T time can be applied to estimate the ventilatory efficiency of patients with CHF. Future studies may use the GA-T in conjunction with other functional capacity tests to guide the treatment plan and evaluate the prognosis.

## Introduction

In the evaluation of patients with chronic heart failure (CHF), cardiopulmonary exercise testing (CPET) is often used to establish functional capacity and guide treatment, as it provides some indices that are associated with the physical fitness and clinical prognosis of affected individuals [1]. Some of the ventilatory variables obtained in CPET that have this purpose are the peak oxygen uptake (VO_2peak_) and the ventilatory efficiency, which is measured using indices such as the ventilatory equivalent for oxygen and the ventilatory equivalent for carbon dioxide [2]. The latter represents the regression slope relating minute ventilation to carbon dioxide output (VE/VCO_2_ slope). The significant increase in VE/VCO_2_ slope in patients with CHF can be explained by changes in the ventilation/perfusion ratio (V/Q ratio), phenomena at the alveolar–capillary membrane, and stimulation of muscle metaboreceptors [3, 4]. In routine clinical practice, however, there are limitations to applying CPET, such as the high cost of equipment and the need for complex infrastructure with trained professionals [5]. These characteristics highlight the importance of using tests that indirectly assess functional exercise capacity in patients with CHF.

The 6-min walk test (6MWT) and the shuttle walk test, which measure the distance travelled along a given route, stand out among the field tests that provide an indirect measure of functional exercise capacity in patients with CHF [6, 7]. Considering the need to involve other body structures to better represent activities of daily living (ADLs), Skumlien et al. [8] proposed a test that involves multitasking that is similar to ADLs, called the Glittre-ADL test (GA-T). Considering the practicality, low cost, and range of body functions involved in GA-T and the importance of ventilatory efficiency measured by the VE/VCO_2_ slope for prognostic evaluation of patients with CHF, we sought to test for an association between the GA-T time and VE/VCO_2_ slope measured by telemetry.

## Main text

### Methods

This cross-sectional study evaluated eight (of 12 eligible) patients with CHF regularly managed at the Coração Valente Heart Failure Clinic of the Fluminense Federal University, Niterói, Brazil. The inclusion criteria were patients with New York Heart Association (NYHA) class II–III and age > 55 years. The following exclusion criteria were applied: decompensated CHF (Stevenson’s class C); history of recent acute myocardial infarction; uncontrolled arrhythmia; report of previous or current smoking; presence of musculoskeletal or neurological diseases that limited movement; and history of hospitalization in the last three months. The protocol was previously approved by the Research Ethics Committee of the Augusto Motta University Centre under number 1.631.435, and all participants signed the consent form.

**Fig. 1:**
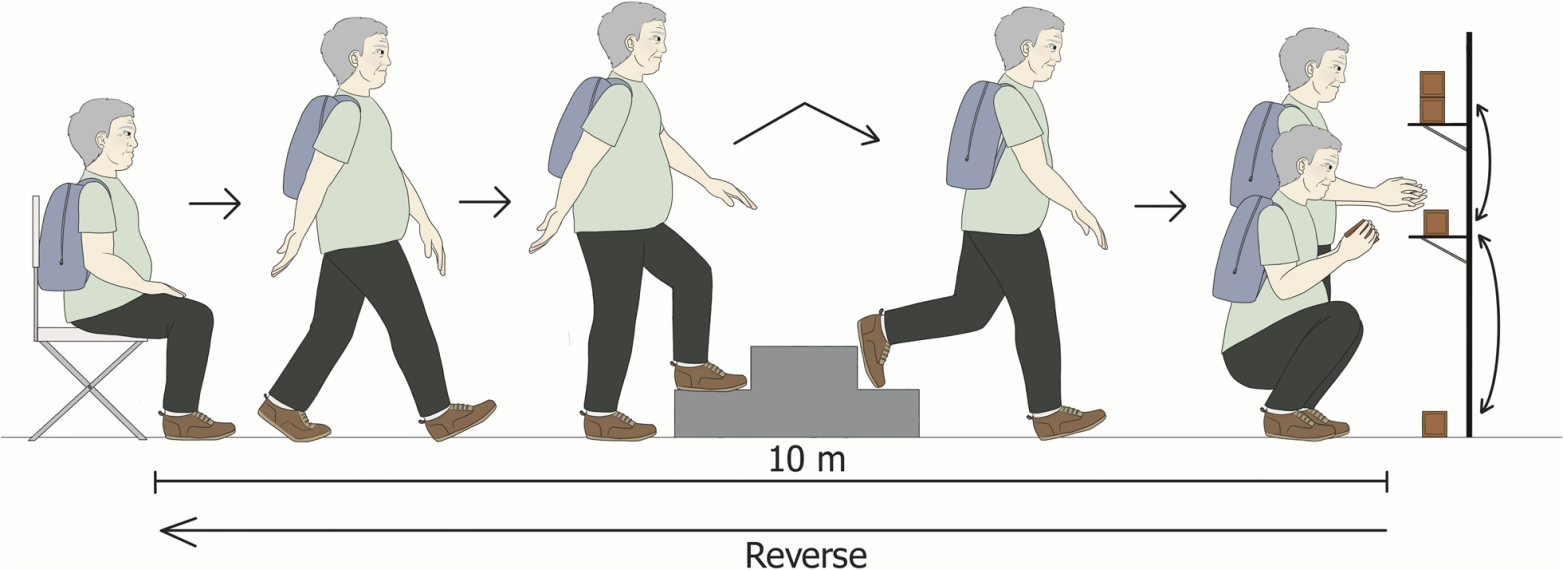
Tasks involved in the Glittre-ADL test. A chair was placed at one end of a corridor, and the patient was instructed to sit down on the chair before beginning the tasks. A bookcase with two shelves was placed at the opposite end of the corridor, and three objects weighing 1 kg each were placed on the shelves. Between the chair and the bookcase, there was an object consisting of two stairsteps. The participant wore a backpack with a certain weight (2.5 kg for women and 5 kg for men). Then, the participant was asked to stand up from the chair and travel towards the shelves, travel up and down the steps towards the other end of the corridor, and move the objects from the highest to the lowest shelf and then from the lowest shelf to the floor. Immediately after that, the individual had to move the objects from the floor to the lowest shelf and then from the lowest shelf to the highest shelf. After finishing these tasks, he/she had to return by climbing up and down the steps and sit on the chair. The participant had to repeat the entire route four more times

In addition to the anamnesis and physical examination, the participants were subjected to analysis of functional exercise capacity by means of GA-T coupled with a metabolic gas analyser to obtain the ventilatory variables through telemetry. GA-T was performed as described by Skumlien et al. [8] on a 10-m route (Fig. 1). Before the beginning of the test, a metabolic gas analyser was coupled to the patient to measure the ventilatory variables through telemetry (VO2000, ErgoMET 13 software, MedGraphics, Brazil). Immediately before and after the test, the Borg Rating of Perceived Exertion (RPE) scale (fatigue and dyspnoea) was used, and pulse oximetry, blood pressure, and peripheral oxygen saturation were measured [9, 10]. The VO_2peak_ value considered for analysis was the value at the end of the test, while the VE/VCO_2_ slope value was the mean of all values obtained during GA-T.

The data were analysed in IBM SPSS Statistics 23 software. The results are expressed as median (minimum – maximum) or frequency (percentage). The difference between the medians of a continuous variable before and after GA-T was assessed by the Wilcoxon test. The association between the variables was calculated as the Spearman correlation coefficient (r_s_). The significance level adopted was 5%.

### Results

Of the 12 patients who agreed to participate in the study, four were excluded due to poor perfusion associated with pulmonary congestion (Stevenson’s class C). The final sample consisted of eight patients, of whom four were female, and the median age was 67 (57–75) years. The comorbidities present were diabetes mellitus (*n* = 2), previous acute myocardial infarction (*n* = 2), coronary artery disease (*n* = 1), chronic renal failure (*n* = 1), and dyslipidaemia (*n* = 1). The characteristics of the sample in terms of demographic data, CHF characteristics, drug use, and CPET results are shown in Table 1.

**Table 1:** Characteristics of the sample and cardiopulmonary exercise testing results

Variable	Value
Demographic data	
Age (years)	67 (57–75)
Gender (male)	4 (50%)
Weight (kg)	72.5 (42.3–100.2)
Height (cm)	159 (145–170)
BMI (kg/m^2^)	28.2 (19.6–33.1)
Characteristics of CHF	
NYHA class II	6 (75%)
NYHA class III	2 (25%)
LVEF (%)	61.2 (47–72.6)
Preserved ejection fraction	5 (62.5%)
Reduced ejection fraction	3 (37.5%)
BNP (pg/mL)	99.1 (85–118)
Current drugs	
Diuretic	8 (100%)
ACEI	6 (75%)
β-blocker	6 (75%)
MRA	3 (37.5)
Sacubitril/valsartan	2 (25)
Anticoagulant	4 (50%)
CPET results	
VO_2peak_ (mL/kg/min)	15.6 (13.1–16.5)
VE/VCO_2_ slope	22.3 (24.1–19.2)
HR_max_ (beats/min)	138 (97–154)
Oxygen pulse (mL/beat)	9.52 (7.40–12.9)
SBP during peak exercise (mmHg)	162 (114–195)
DBP during peak exercise (mmHg)	93 (65–128)
RER at rest	0.85 (0.81–0.90)
RER at peak exercise	1.09 (0.95–1.25)

*BMI* body mass index, *CHF* chronic heart failure, *LVEF* left ventricular ejection fraction, *BNP* B-type natriuretic peptide, *NYHA* New York Heart Association, *ACEI* angiotensin-converting enzyme inhibitors, *MRA* mineralocorticoid receptor antagonist, *CPET* cardiopulmonary exercise testing, *VO*_*2peak*_ peak oxygen uptake, *VE/VCO*_*2*_* slope* the relation between the regression slope relating minute ventilation to carbon dioxide output, *HR*_*max*_ maximum heart rate, *SBP* systolic blood pressure, *DBP* diastolic blood pressure, *DBP* diastolic blood pressure, *RER* respiratory exchange ratio. The results are expressed as the median (minimum–maximum) or a number (%)

The median GA-T execution time in the studied patients was 4 min 39 s (3 min 29 s–5 min 53 s). Statistically significant variations were identified between the pre- and post-GA-T measurements for the following variables: RPE scale (fatigue) [1.5 (1–3) vs. 4 (3–7), *P* = 0.011]; RPE scale (dyspnoea) [2 (0–9)3 vs. (1–10), *P* = 0.039]; heart rate [72 (59–95) vs. 114 (69–123) bpm, *P* = 0.012]; systolic blood pressure [125 (100–162) vs. 158 (110–190) mmHg, *P* = 0.027]; and diastolic blood pressure [65 (60–100) vs. 91 (60–120) mmHg, *P* = 0.043].

Finally, we evaluated the associations between total GA-T time, the RPE scale (fatigue and dyspnoea), left ventricular ejection fraction (LVEF), and CPET variables. In this analysis, the only significant correlations were between VE/VCO_2_ and total GA-T time and between the VE/VCO_2_ slope and RPE scale (fatigue) (Table 2). In addition, no significant correlation was found between B-type natriuretic peptide levels and the CPET results.

**Table 2:** Spearman correlation coefficients between ventilatory variables and total Glittre-ADL test time, perception of effort, and ejection fraction

Variables	Total GA-T time	RPE scale (fatigue)	RPE scale (dyspnoea)	LVEF
VO_2peak_	− 0.429	− 0.110	− 0.390	0.192
VE/VCO_2_ slope	0.714^*^	0.761^*^	0.610	− 0.443
HR_max_	0.381	0.210	0.154	− 0.224
Oxygen pulse	− 0.326	− 0.296	− 0.127	0.176
SBP during peak exercise	0.252	0.163	0.219	− 0.164
DBP during peak exercise	0.173	0.168	0.150	− 0.123
RER at rest	0.125	0.173	0.155	− 0.121
RER during peak exercise	0.214	0.120	0.111	− 0.247

*GA-T* Glittre-ADL test, *RPE* Borg rating of perceived exertion, *LVEF* left ventricular ejection fraction, *VO*_*2peak*_ peak oxygen uptake, *VE/VCO*_*2*_* slope* the relation between the regression slope relating minute ventilation to carbon dioxide output, *HR*_*max*_ maximum heart rate, *SBP* systolic blood pressure, *DBP* diastolic blood pressure, *DBP* diastolic blood pressure, *RER* respiratory exchange ratio

^*^P < 0.05

### Discussion

The main findings of the present study were that in patients with CHF, there are significant associations between VE/VCO_2_ slope and functional exercise capacity assessed through the GA-T time and between VE/VCO_2_ slope and exercise tolerance measured through the RPE scale for fatigue. To date, there is little evidence on the association between the total time taken to perform the multitasking GA-T and the ventilatory variables in patients with CHF. The knowledge of these relationships is important because they can suggest the clinical prognosis and the evolution of pharmacological and nonpharmacological treatment.

Functional capacity tests with indirect measures are widely used in patients with CHF because they are inexpensive and very safe and reflect energy expenditure during the execution of ADLs [11]. Valadares et al. [12] used the GA-T to evaluate the functional capacity of patients with CHF with NYHA functional classes III and IV. They sought to correlate the total GA-T time with the distance travelled in the 6MWT and found a significant correlation between the variables, demonstrating the applicability of the GA-T in patients with CHF. Skumlien et al. [8] and Valadares et al. [12] demonstrated the applicability of the GA-T to more severe and symptomatic patients, but our results suggest that the GA-T also has good applicability in less symptomatic patients (NYHA classes II and III) given the strong correlation observed between the RPE scale for fatigue and ventilatory efficiency.

According to Zotter-Tufaro et al. [13], a lower performance in the 6MWT reflects worse prognosis and higher risk of mortality in patients with CHF, which corroborates the findings of the present study, since a longer GA-T execution time was associated with a higher VE/VCO_2_ slope value, and this ventilatory variable is a predictor of the prognosis of CHF. In this context, it is possible to consider GA-T another test of functional exercise capacity to evaluate the evolution of CHF, considering that the VE/VCO_2_ slope is correlated with ventilatory efficiency. This association can be explained by the fact that a greater capacity to exercise is responsible for better ergoreflex control, greater mitochondrial activity, greater cardiac output, and greater V/Q ratio compatibility, which are the main factors that, if altered, affect the VE/VCO_2_ ratio in patients with CHF [14]. The improvement in cardiac output facilitates the transport of oxygen to skeletal muscle, which increases ventilatory control, thus providing a respiratory pattern that favours the V/Q ratio and, consequently, the VE/VCO_2_ slope [15]. These findings may have implications for the use of GA-T to evaluate the effects of rehabilitation programmes on ventilatory efficiency, since the improvement in the functions of the upper limbs, lower limbs, and trunk may reduce the GA-T execution time.

VO_2peak_ was initially proposed as another variable of prognostic value for patients with CHF [16], but it is strongly influenced by several factors such as sex, age, muscle conditioning, and comorbidities and has therefore been analysed together with the VE/VCO_2_ slope for better interpretation of functional capacity and prognosis in clinical practice [5]. In fact, we did not observe a significant association between total GA-T time and VO_2peak_. In contrast, Palau et al. [17] observed a correlation between the distance covered in the 6MWT and VO_2peak_ in patients with CHF. This difference can be explained in part by the way in which the tests are performed: The activities of the upper and lower limbs alternate in GA-T, in which there are periods of more and less exertion depending on the different overloads imposed by the exercise, whereas in 6MWT, ambulation is the only factor that increases cardiac overload as a function of time. Thus, it is possible to infer that the evaluation of VO_2peak_ in GA-T promotes an interpretation different from that obtained in 6MWT due to the scope of the multitasking and the constant alternation of the overload.

## Limitations

The main limitations were the fact that the GA-T was applied only once, the small sample size, and the absence of a control group. Despite the small sample size, this study offers promising preliminary results on the association between functional exercise capacity measured by GA-T and the VE/VCO_2_ slope in patients with CHF. Despite these limitations, this study can serve as a basis for future trials with more participants to evaluate the multiple tasks of the GA-T together with telemetry. Patients can be separated into subgroups based on the aetiology of CHF, functional class, and LVEF for more detailed outcomes regarding functional exercise capacity in this population. Based on longitudinal studies, prognostic cut-off points can be established from the VO_2peak_ and VE/VCO_2_ slope values obtained.

## Data Availability

Data and materials are available from the corresponding author upon reasonable request.

## Abbreviations

6MWT: 6-Min walk test
ADL: Activities of daily living
CHF: Chronic heart failure
CPET: Cardiopulmonary exercise testing
GA-T: Glittre-ADL test
RPE: Borg Rating of Perceived Exertion
LVEF: Left ventricular ejection fraction
NYHA: New York Heart Association
UFF: Fluminense Federal University
UNISUAM: Augusto Motta University Center
VE/VCO_2_ slope: The relation between the regression slope relating minute ventilation to carbon dioxide output
VO_2peak_: Peak oxygen uptake
V/Q ratio: Ventilation/perfusion ratio
